# A Rare Case of Takayasu Arteritis and Aortic Aneurysm in a Male Patient

**DOI:** 10.7759/cureus.94910

**Published:** 2025-10-19

**Authors:** André Pereira, Cristina Silva, Sara Freitas, Glória Alves, Jorge Cotter

**Affiliations:** 1 Internal Medicine, Unidade Local de Saúde Alto Ave, Guimarães, PRT

**Keywords:** ascending aortic aneurysm, carotid artery occlusion, fever of unknown origin, immunosuppressive therapy, large-vessel vasculitis, takayasu arteritis

## Abstract

Takayasu arteritis (TA) is a rare large-vessel vasculitis typically affecting young women. Fever of unknown origin (FUO) can be an early manifestation, especially in atypical demographic groups, delaying diagnosis. A 49-year-old male presented with a month-long history of daily intermittent fevers, night sweats, weight loss, anorexia, and dry cough. Laboratory studies showed anemia, elevated inflammatory markers, and hyperferritinemia. Extensive infectious and autoimmune and workup was negative. During oncologic screening, colonoscopy identified a rectal intramucosal adenocarcinoma, which was completely resected. An 18F-fluorodeoxyglucose positron emission tomography/computed tomography (18F-FDG PET/CT) revealed increased uptake in the thoracic aorta, brachiocephalic trunk, and bilateral carotid and subclavian arteries. Transesophageal echocardiography confirmed a 55-mm ascending aortic aneurysm. The patient later developed pericarditis, upper limb claudication, and right internal carotid artery occlusion, fulfilling the diagnostic criteria for TA. He was treated with corticosteroids and methotrexate, followed by ascending aorta replacement. Follow-up PET/CT showed resolution of inflammation, with sustained remission.

This case illustrates the importance of considering TA in FUO. An 18F-FDG PET/CT can be pivotal in diagnosis, enabling early treatment and preventing vascular complications.

## Introduction

Takayasu arteritis (TA) is a rare, chronic large-vessel vasculitis that primarily affects the aorta and its major branches. It occurs in approximately one to two individuals per million each year, with a marked female predominance and typical onset before the age of 40 [1,2] . Characterized by inflammation of the arterial wall, the disease may lead to narrowing, occlusion, or aneurysm formation, ultimately resulting in end-organ ischemia [2,3].

In its early stages, TA often presents with nonspecific systemic symptoms such as fever, weight loss, malaise, and elevated inflammatory markers [2,4]. These features frequently overlap with infectious, autoimmune, or neoplastic conditions, creating a diagnostic challenge, particularly when the disease occurs in atypical demographics such as middle-aged men [3,4]. Fever of unknown origin (FUO) may even represent the initial manifestation, further delaying recognition and management [3].

Advances in imaging, particularly with 18F-fluorodeoxyglucose positron emission tomography/computed tomography (18F-FDG PET/CT), have significantly improved early detection of vascular inflammation, even before structural changes become apparent [4,5]. The 2023 EULAR recommendations identify 18F-FDG PET/CT as a key diagnostic and monitoring tool in suspected large-vessel vasculitis [4].

We report the case of a middle-aged man who presented with FUO and was ultimately diagnosed with TA complicated by an ascending aortic aneurysm. This case highlights the importance of considering large-vessel vasculitis in the differential diagnosis of FUO, emphasizes the diagnostic role of 18F-FDG PET/CT, and demonstrates the value of a multidisciplinary approach to management.

## Case presentation

A 49-year-old autonomous white male patient presented with a one-month history of daily intermittent fevers (maximum 38-39°C), night sweats, chills, and unintentional 6.6% weight loss. He also reported anorexia and a dry, irritative cough. There was no relevant past medical history, recent travel, animal contact, or ingestion of untreated water.

On admission, the patient was febrile (39.1°C) with a blood pressure of 144/86 mmHg in both arms, heart rate of 96 bpm, respiratory rate of 17 breaths per minute, and oxygen saturation 98% on room air. The remainder of the physical examination was unremarkable. Laboratory findings included normocytic normochromic anemia, elevated erythrocyte sedimentation rate (ESR), markedly increased C-reactive protein (CRP), and hyperferritinemia, with normal renal function and coagulation parameters (Table 1).

**Table 1:** Blood test results performed at hospital admission. APTT, activated partial thromboplastin time; CRP, C-reactive protein; ESR, erythrocyte sedimentation rate; INR, international normalized ratio; MCHC, mean corpuscular hemoglobin concentration; MCV, mean corpuscular volume; TIBC, total iron-binding Capacity; TSAT, transferrin saturation

Test	Result	Reference range
Hemoglobin (g/dL)	11.2	14.0-18.0
Hematocrit (%)	35.0	41-53
MCV (fL)	90.0	83-103
MCHC (g/dL)	32.0	32.0-36.0
ESR (mm/h)	63	0-12
CRP (mg/L)	146	<3.0
Urea (mg/dL)	24	15-39
Creatinine (mg/dL)	0.76	0.70-1.30
Sodium (mEq/L)	139	135-136
Potassium (mEq/L)	4.28	3.5-5.1
Chloride (mEq/L)	103	95-105
Calcium (mg/dL)	8.9	8.3-10.6
Phosphorus (mg/dL)	3.8	2.5-4.9
Magnesium (mg/dL)	2.55	1.6-2.6
Serum iron (µg/dL)	27	65-175
TIBC (µg/dL)	233	250-425
Transferrin (mg/dL)	178	215-365
TSAT (%)	11.6	15-50
Ferritin (ng/mL)	1131.1	22-322
Prothrombin time (sec)	14.4	8.4-14.4
INR	1.35	–
APTT (sec)	29.4	20.9-34.9

**Figure 1:**
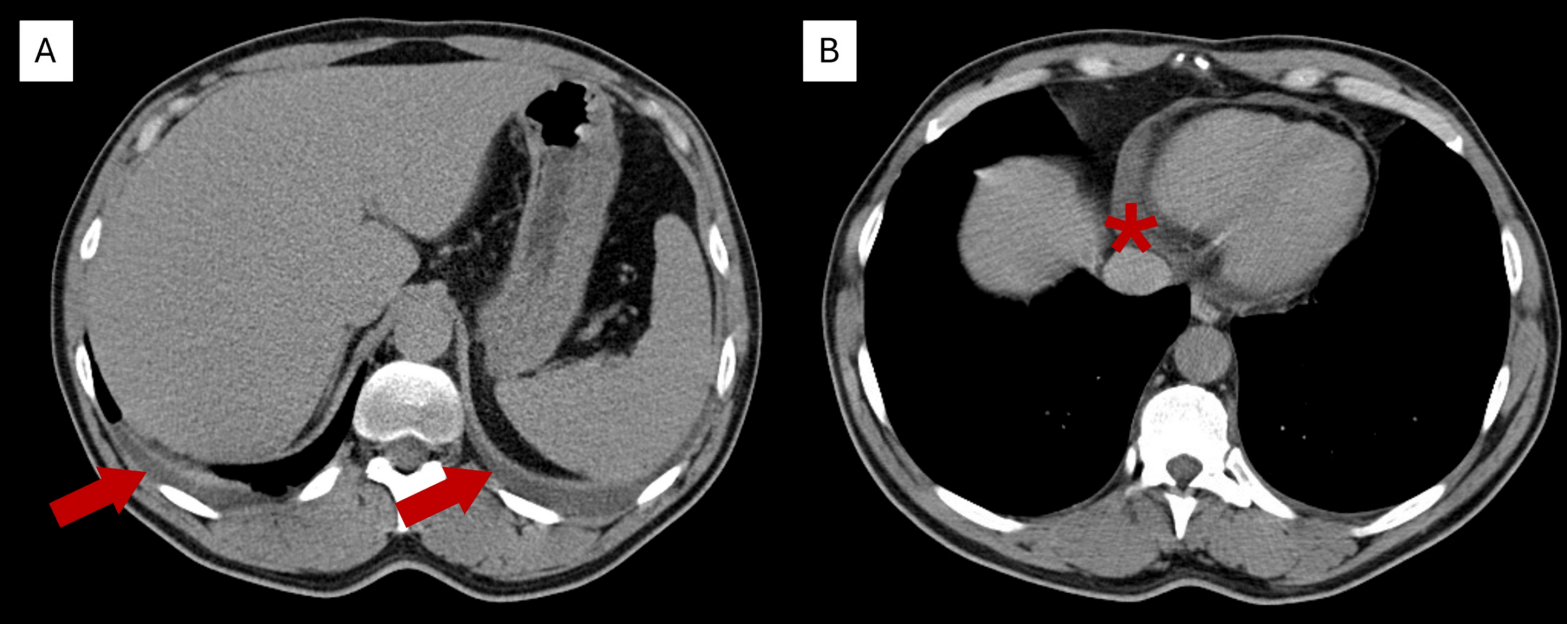
Axial contrast-enhanced CT scan. CT images showing (A) small bilateral pleural effusions (red arrows) and (B) a small pericardial effusion (*), consistent with mild serosal inflammation.

Chest radiography was normal. Contrast-enhanced CT of the chest, abdomen, and pelvis revealed small bilateral pleural and pericardial effusions, mild hepatomegaly with fatty infiltration, and no lymphadenopathy or masses (Figure 1).

The patient was admitted for monitoring and further etiological investigation.

Microbiological studies, including SARS-CoV-2 polymerase chain reaction (PCR), blood and urine cultures, and serologies for human immunodeficiency virus, hepatitis B and C, syphilis, *Legionella*, *Leptospira*, Epstein-Barr virus, cytomegalovirus, and *Toxoplasma* were negative. Ziehl-Neelsen stain of the sputum was also negative.

Autoimmune markers (antinuclear antibodies, rheumatoid factor, complement, and immunoglobulins) were within normal limits, reducing the likelihood of connective tissue disease. The prostate-specific antigen was also normal (Table 2).

**Table 2:** Additional laboratory investigation. ANA, antinuclear antibodies; C3, complement component 3; C4, complement component 4; IgA, immunoglobulin A; IgG, immunoglobulin G; IgM, immunoglobulin M; PSA, prostate-specific antigen

Test	Result	Reference range
ANA	Negative	–
Rheumatoid factor (IU/mL)	<9.7	<20
C3 (mg/dL)	142	82-170
C4 (mg/dL)	29.7	12-36
IgA (mg/dL)	185	40-350
IgG (mg/dL)	777	650-1600
IgM (mg/dL)	78.1	50-350
PSA (ng/mL)	3.7	0.0-4.0

**Figure 2:**
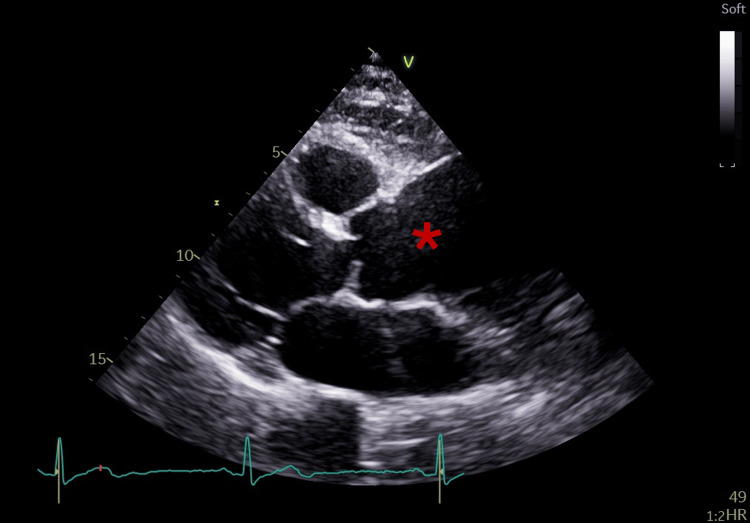
Transthoracic echocardiogram. Transthoracic echocardiography demonstrating a markedly dilated ascending aorta, consistent with ascending aortic aneurysm (*).

Echocardiography on day 3 showed preserved biventricular function, a 5.6-cm ascending aortic aneurysm, mild basal septal thickening, and no vegetations (Figure 2). Thyroid, prostate, inguinal, and axillary ultrasounds were normal.

Five days after admission, bone-marrow analysis was performed to exclude hematologic malignancy or occult infection as causes of persistent FUO; results were unremarkable (Tables 3, 4).

**Table 3:** Bone marrow smears. Bone marrow shows trilinear hematopoiesis with preserved maturation, no excess blasts, and no dysplasia.

Cell Line/Parameter	Result
Blasts	1.7%
Promyelocytes	1.3%
Myelocytes	5.1%
Neutrophilic metamyelocytes	7.5%
Eosinophilic metamyelocytes	0.3%
Neutrophils	47.5%
Eosinophils	1.7%
Basophils	0.5%
Lymphocytes	16.5%
Monocytes	3.7%
Plasma cells	0.1%
Proerythroblasts	0.3%
Basophilic erythroblasts	0.5%
Polychromatic erythroblasts	3.0%
Orthochromatic erythroblast	10.3%
Myeloid to erythroid ratio	4.5

**Table 4:** Bone marrow immunophenotyping. No immunophenotypic abnormalities were identified that would suggest a lymphoproliferative disease.

Parameter	Result
Lymphocyte subsets	
T lymphocytes	76%
B lymphocytes	12%
NK cells	12%
T-cell subsets	
CD4+ T cells	78% of T cells
CD8+ T cells	19% of T cells
CD4/CD8 ratio	4.1
B-cell markers	
CD5+ B cells	6% of B cells
CD10+ B cells	1% of B cells
Kappa light chain	61% of B cells
Lambda light chain	39% of B cells
Kappa/lambda ratio	1.6
B-cell immunophenotype	
CD19	100% of B cells
CD20	100% of B cells
CD45	100% of B cells
Kappa Ig (surface)	61%
Lambda Ig (surface)	39%
T-cell immunophenotype	
CD3	100% of T cells
CD4	78%
CD5	99%
CD8	19%
CD45	100%
CD56	3% of T cells

**Figure 3:**
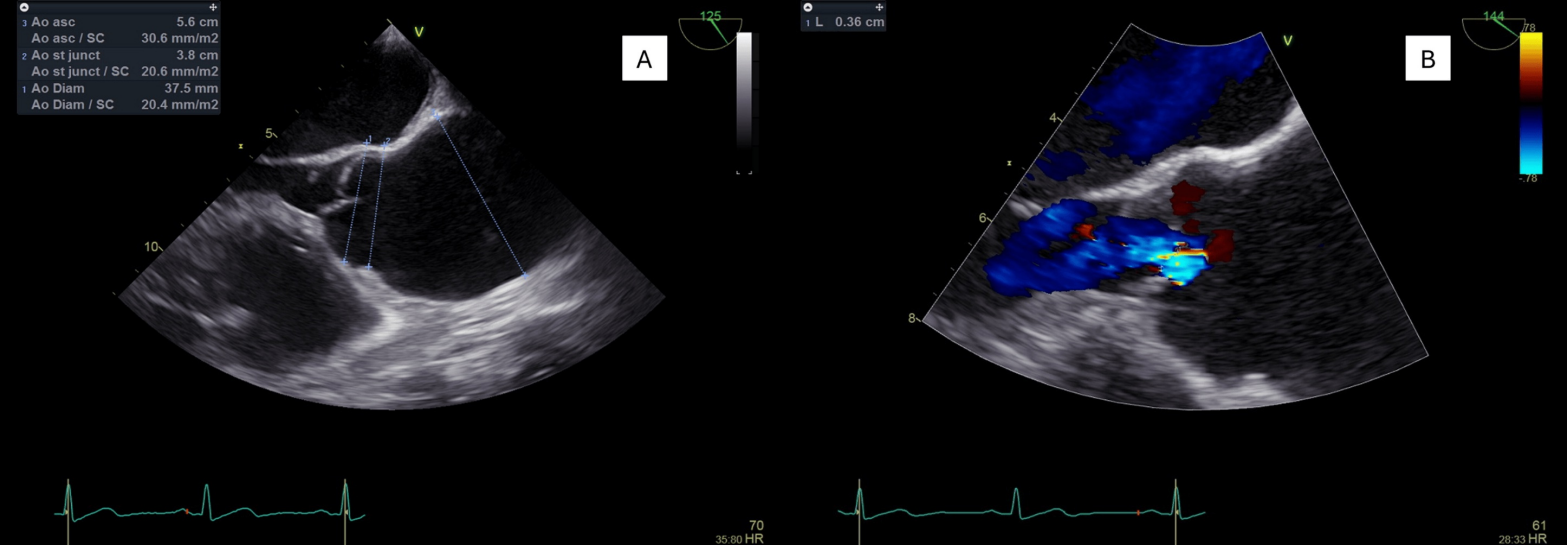
Transesophageal echocardiogram. Transesophageal echocardiography showing (A) ascending aortic aneurysm with 5.6 cm of maximum diameter and (B) Doppler showing central aortic regurgitation across the aortic valve.

**Figure 4:**
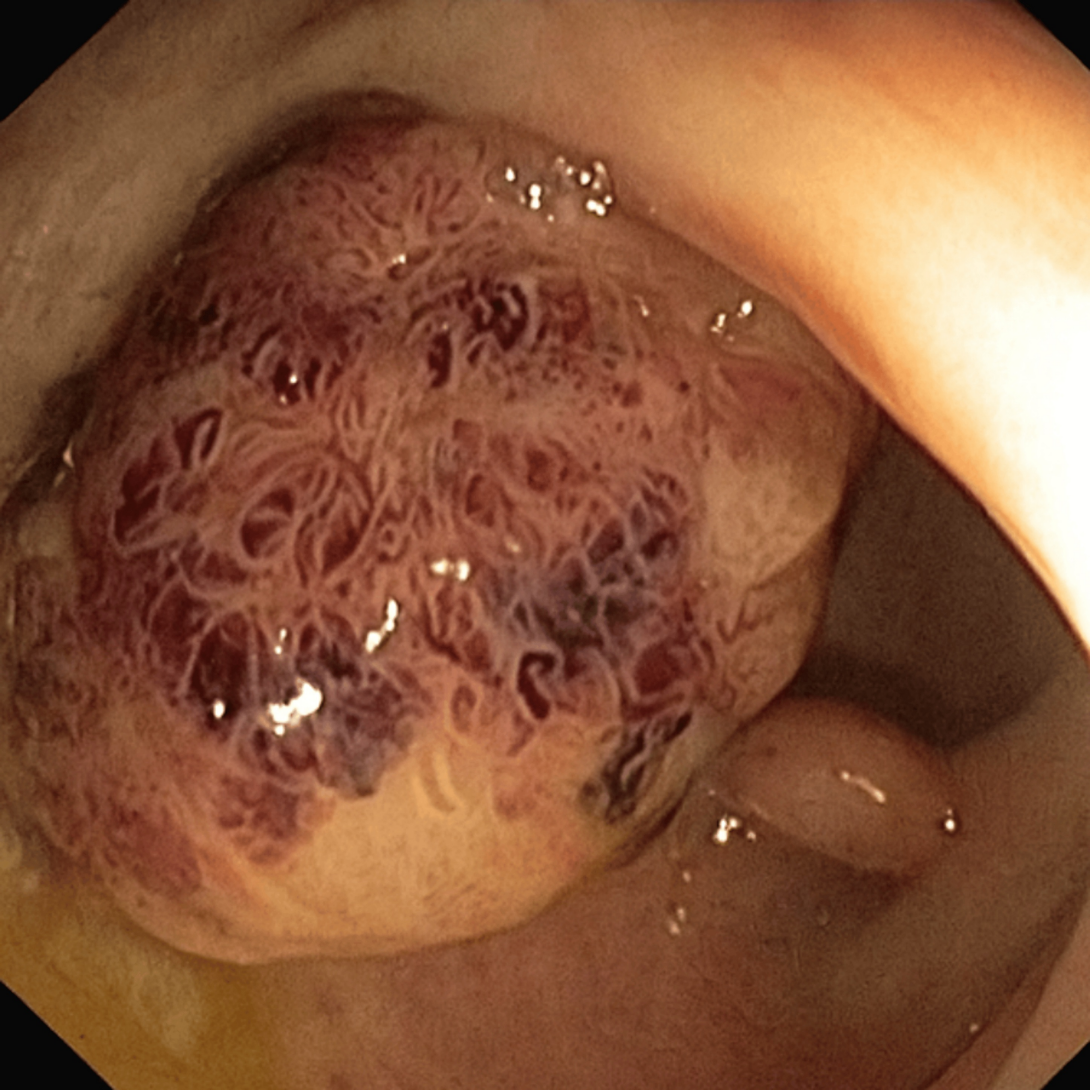
Colonoscopy. Colonoscopy revealing a 25-mm pedunculated polyp in the proximal rectum, resected with diathermic snare; histology was consistent with intramucosal adenocarcinoma.

On day 9, transesophageal echocardiography revealed no additional abnormalities beyond the aneurysm (Figure 3). On day 16, colonoscopy identified a 25-mm pedunculated polyp in the proximal rectum (Figure 4), which was resected completely. Histology confirmed intramucosal adenocarcinoma. Following multidisciplinary discussion, endoscopic surveillance was chosen in coordination with gastroenterology.

**Figure 5:**
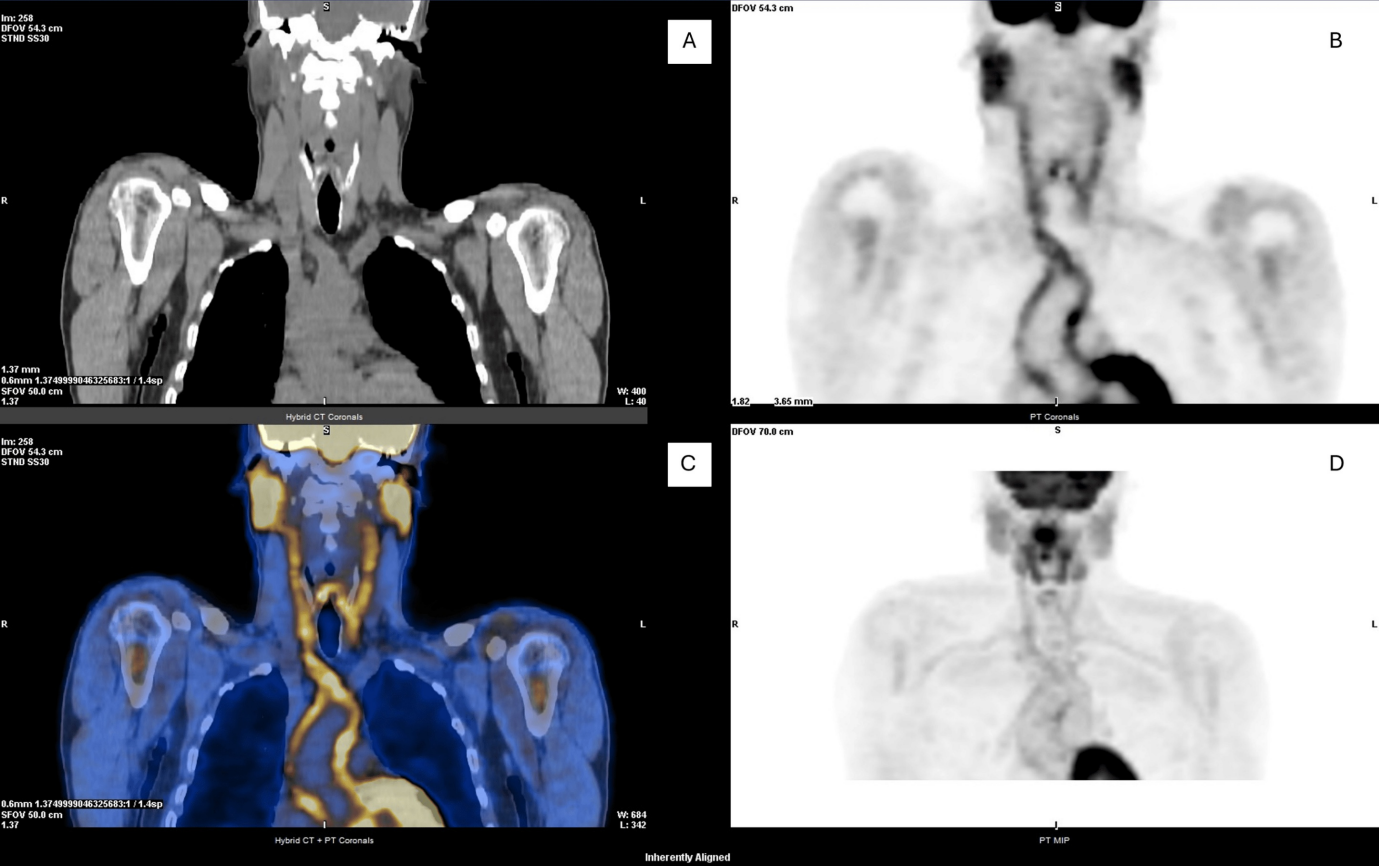
18F-FDG PET/CT imaging. Corresponding CT (A), PET (B), fused PET/CT (C), and PET MIP (D) images showing increased FDG uptake in the thoracic aorta, brachiocephalic trunk, and bilateral carotid arteries, consistent with active large-vessel vasculitis. 18F-FDG, 18F-fluorodeoxyglucose; CT, computed tomography; MIP, maximum intensity projection; PET, positron emission tomography

Persistent fever and inflammatory markers prompted an 18F-FDG PET/CT on day 20, showing increased uptake in the thoracic aorta, brachiocephalic trunk, and bilateral carotid and subclavian arteries, consistent with inflammatory involvement (Figure 5).

Despite extensive evaluation, the underlying cause of FUO could not be clearly established. The patient remained hemodynamically stable but continued to experience one to two febrile peaks daily and was discharged for outpatient follow-up.

**Figure 6:**
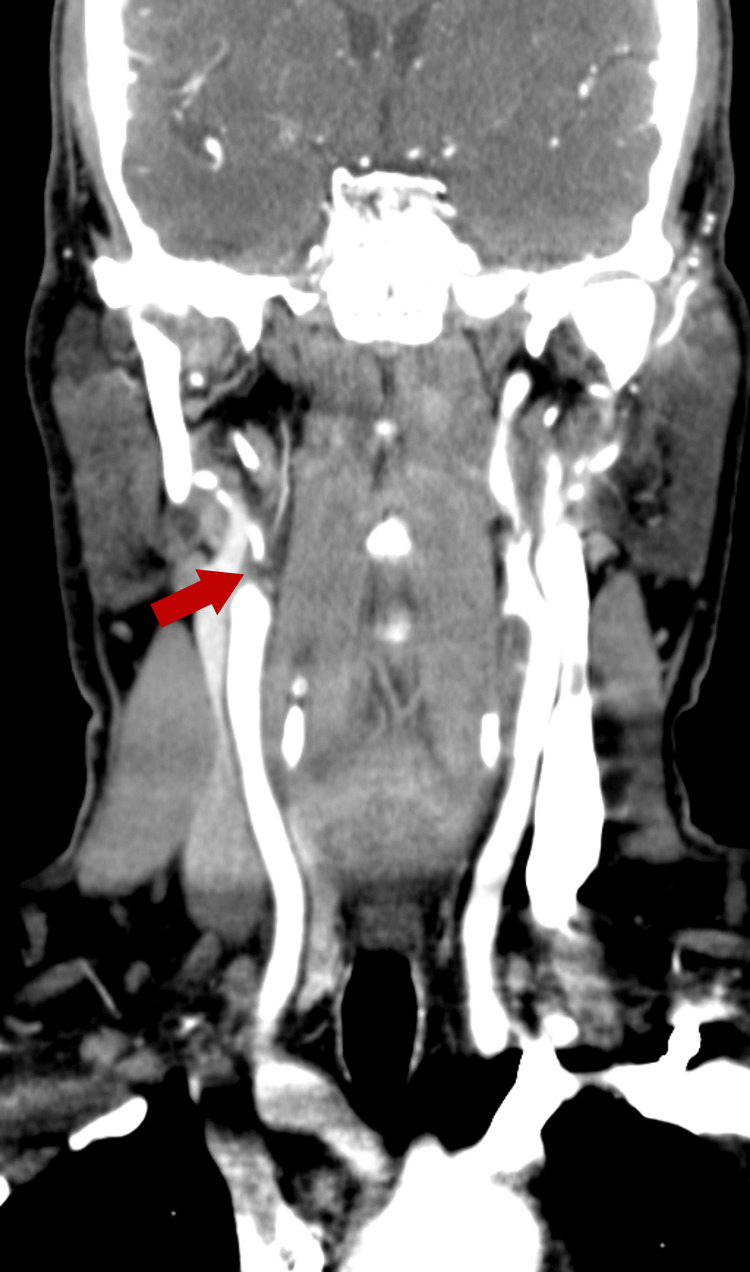
CT angiography. CT angiography demonstrating near-complete occlusion of the right internal carotid artery distal to the carotid bulb, with residual filiform flow (red arrow).

Around one month after hospitalization, he developed intermittent upper-limb claudication with a 10 mmHg systolic blood pressure difference between arms (right 145/76 mmHg; left 155/79 mmHg). The right radial pulse was weaker than the left. CT angiography revealed near-complete occlusion of the right internal carotid artery distal to the carotid bulb (Figure 6). Based on these findings, a diagnosis of TA was established.

**Figure 7:**
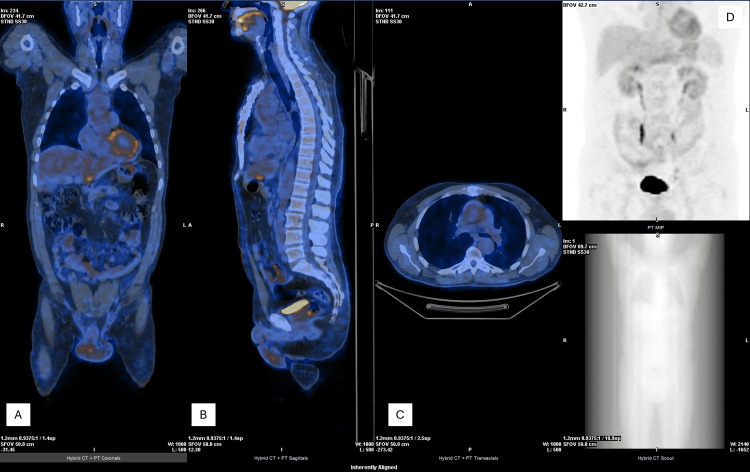
18F-FDG PET/CT imaging. Coronal (A), sagittal (B), axial (C), and MIP (D) PET/CT images showing no residual inflammatory uptake, consistent with metabolic remission. 18F-FDG, 18F-fluorodeoxyglucose; CT, computed tomography; MIP, maximum intensity projection; PET, positron emission tomography

Immunosuppressive therapy with oral corticosteroids (prednisolone 40 mg/day) was initiated and later tapered. After six months, follow-up 18F-FDG PET/CT demonstrated no significant residual inflammatory activity (Figure 7). Corticosteroids were discontinued, and methotrexate (10 mg weekly) was started.

After approximately one year of immunosuppressive therapy, with imaging evidence of controlled disease, the patient underwent elective ascending aorta replacement. The subsequent clinical course was favorable, and no recurrence of constitutional symptoms during follow-up.

## Discussion

TA is a rare, chronic, granulomatous vasculitis that primarily affects the aorta and its major branches. Although it classically occurs in young women under 40, atypical presentations, particularly in men and older adults, pose significant diagnostic challenges and often lead to delays in recognition [2,3]. In this context, our patient, a 49-year-old man, represents an uncommon demographic, underscoring the need for clinical vigilance when evaluating persistent FUO with elevated inflammatory markers.

In its early phase, TA commonly manifests with nonspecific systemic symptoms such as fever, night sweats, weight loss, and fatigue that may precede vascular complications by months or years [2,4]. These prodromal features, as seen in our patient, frequently resemble infections, autoimmune diseases, or malignancies, complicating the diagnostic pathway. FUO can be the initial manifestation in up to 20-30% of TA cases [3]. In this case, diagnostic interpretation was further confounded by the incidental discovery of an intramucosal rectal adenocarcinoma, which initially appeared to explain the systemic inflammatory response. This overlap delayed recognition of the underlying vasculitic process until vascular imaging clarified the diagnosis.

The coexistence of TA and adenocarcinoma in this case also raises the possibility of a biological association between large-vessel vasculitis and malignancy. Although TA is typically considered idiopathic, paraneoplastic mechanisms involving immune cross-reactivity, immune-complex deposition, or tumor-derived cytokine release have been proposed, particularly in adenocarcinomas [6,7]. While causality cannot be confirmed, this temporal relation supports the hypothesis that neoplastic processes may occasionally trigger or modulate vascular inflammation. This potential relationship merits further investigation and consideration.

Recognizing these diagnostic complexities highlights the essential role of imaging in establishing the diagnosis of TA. Modern imaging modalities allow for the detection of inflammatory activity before irreversible structural vessel damage occurs. Among these, 18F-FDG PET/CT has emerged as a highly sensitive technique for identifying early vascular inflammation [1,4,5]. In this patient, 18F-FDG PET/CT demonstrated diffuse hypermetabolic activity involving the thoracic aorta, brachiocephalic trunk, and supra-aortic branches, confirming active vasculitis and guiding appropriate immunosuppressive therapy.

Integration of laboratory and imaging data was crucial in narrowing the differential diagnosis. The normalization of inflammatory markers following treatment paralleled the resolution of vascular FDG uptake, reinforcing the diagnosis of active vasculitis rather than infection or neoplastic fever.

Recent evidence supports the diagnostic and monitoring value of 18F-FDG PET/CT. Meta-analyses report a sensitivity approaching 95-100% and a specificity around 75%, outperforming conventional imaging for early disease detection [3,5]. Quantitative 18F-FDG PET/CT parameters, such as the maximum standardized uptake value (SUVmax), correlate with histopathologic activity and may help predict therapeutic response [1,4].

Our case aligns with current EULAR recommendations and highlights the dual role of 18F-FDG PET/CT in both the diagnosis and follow-up of TA [8]. Persistent vascular uptake after treatment has been linked to a higher risk of relapse and progressive vascular injury [1,5]. In our case, follow-up 18F-FDG PET/CT demonstrated complete metabolic remission, supporting corticosteroid tapering and transition to methotrexate maintenance therapy. These findings emphasize the value of 18F-FDG PET/CT in guiding personalized management based on disease activity.

Despite effective disease control, our patient developed an ascending aortic aneurysm measuring 55 mm, a recognized yet serious complication of TA. This reflects chronic transmural inflammation and progressive weakening of the arterial wall characteristic of the disease [2]. Aneurysms occur in approximately 20-30% of TA patients, and involvement of the ascending aorta is particularly associated with adverse long-term outcomes [3].

Management of TA is inherently multimodal, combining immunosuppressive therapy to control inflammation with surgical or endovascular repair when structural complications arise. High-dose corticosteroids remain the cornerstone of initial treatment and induce remission in most patients; however, relapse or steroid dependence develops in up to 50% [3]. For this reason, steroid-sparing agents such as methotrexate, azathioprine, or biologic therapies are frequently introduced [2]. In this case, the combined use of corticosteroids and methotrexate achieved metabolic remission and allowed for elective ascending aortic replacement, an approach consistent with contemporary best practices.

## Conclusions

TA may present as FUO without the classic ischemic signs and in atypical demographics such as middle-aged men. Furthermore, 18F-FDG PET/CT is a crucial diagnostic tool that enables early detection of large-vessel inflammation and facilitates both diagnosis and therapeutic monitoring. Prompt initiation of immunosuppressive therapy, combined with timely surgical management of vascular complications, can lead to favorable clinical outcomes.

This case also illustrates the potential coexistence of TA with solid-organ malignancies such as rectal adenocarcinoma, raising the possibility of a paraneoplastic association. Although a direct causal link remains speculative, the temporal overlap underscores the importance of evaluating for underlying neoplastic processes in atypical or late-onset presentations of vasculitis. Further studies are warranted to clarify potential paraneoplastic mechanisms and their implications for disease management and prognosis.
